# Genome Sequence of *Flavobacterium akiainvivens* IK-1^T^, Isolated from Decaying *Wikstroemia oahuensis*, an Endemic Hawaiian Shrub

**DOI:** 10.1128/genomeA.01222-15

**Published:** 2015-10-22

**Authors:** Xuehua Wan, Shaobin Hou, Jennifer A. Saito, Kenneth Y. Kaneshiro, Stuart P. Donachie

**Affiliations:** a Advanced Studies in Genomics, Proteomics and Bioinformatics, University of Hawaii at Manoa, Honolulu, Hawaii, USA; b Center for Conservation Research and Training, Pacific Biosciences Research Center, University of Hawaii at Manoa, Honolulu, Hawaii, USA; c Department of Microbiology, University of Hawaii at Manoa, Honolulu, Hawaii, USA

## Abstract

*Flavobacterium* spp. have been cultivated from diverse aquatic and terrestrial habitats. *F. akiainvivens* IK-1^T^ was cultivated from decaying wood of *Wikstroemia oahuensis*, an endemic Hawaiian shrub. The strain’s genome sequence may provide insights into niche adaptation and evolution of the genus in a mid-ocean archipelago.

## GENOME ANNOUNCEMENT

Among the more than 130 *Flavobacterium* spp., some are implicated in diseases in animals and humans (1–3), while some protect plants against pathogens (4). We report the draft genome sequence of *F. akiainvivens* IK-1^T^, isolated from decaying wood of *Wikstroemia oahuensis*, an endemic Hawaiian flowering shrub (5).

Genomic DNA was isolated with the Wizard Genomic DNA purification kit (Promega, USA), with an additional CTAB extraction step to remove abundant exopolysaccharides. Shotgun and 8-kb paired-end libraries sequenced in a Roche 454 GS FLX+ platform generated 157.1 Mb of shotgun sequences and 64.4 Mb of 8-kb paired-end sequences, providing ~49× genome coverage; 99.4% of all bases were assigned Phred quality scores above Q20, and all scored above Q10 in the ea-utils program (6). Five scaffolds totaling 4,532,192 bp (scaffold *N*_50_ = 4,520,429 bp) were built in Newbler version 2.8. *In silico* gap closure proceeded through shredding 454 reads into 100-bp sequences with 50-bp overlap in EMBOSS (7). BGI GapCloser version 1.12 (http://soap.genomics.org.cn) closed gaps covering ~31 kb; full 454 reads were mapped to scaffolds using bowtie2 (8) to confirm the closed gaps were supported by 454 reads, but ~2.3 kb of gaps remain. The genome’s G+C content is 43.8%.

The genome was annotated in the NCBI Prokaryotic Genome Annotation Pipeline (PGAP) (9), and the Rapid Annotation using Subsystem Technology (RAST) server (10, 11). PGAP identified 3,841 protein-coding genes, 175 pseudogenes, 48 tRNA coding regions, and 9 rRNA coding regions. RAST identified 4,067 protein-coding open reading frames, and 338 subsystems. No flagella or chemotaxis systems were identified by RAST. The former is consistent with the strain’s formal description (5). No phage or prophage regions were identified, although 39 proteins were predicted as similar to conjugative transposons in Bacteroidales by RAST. One CRISPR (clustered regularly interspaced short palindromic repeats) region containing 29 spacers was identified with CRISPRFinder (12, 13). Further manual annotation of the *F. akiainvivens* IK-1^T^ genome and a comparative analysis with those of other *Flavobacterium* spp. and phylum *Bacteroidetes* spp. will shed light on potential habitat-specific traits and genome evolution in the phylum (14).

### Nucleotide sequence accession numbers.

This whole-genome shotgun project has been deposited in DDBJ/EMBL/GenBank under the accession number LIYD00000000. The version described in this paper is the first version, LIYD01000000.
